# GABA and 5-HT Systems Are Involved in the Anxiolytic Effect of Gan-Mai-Da-Zao Decoction

**DOI:** 10.3389/fnins.2018.01043

**Published:** 2019-01-22

**Authors:** Hong-Shu Chen, Li-Jia Gu, Yuan-Xiao Yang, Jian-You Guo

**Affiliations:** ^1^The First Affiliated Hospital of Zhejiang Chinese Medical University, Hangzhou, China; ^2^CAS Key Laboratory of Mental Health, Institute of Psychology, Chinese Academy of Sciences, Beijing, China; ^3^University of Chinese Academy of Sciences, Beijing, China; ^4^Hangzhou Medical College, Hangzhou, China; ^5^Henan Key Laboratory of Zhang Zhongjing Formulae and Herbs for Immunoregulation, Nanyang Institute of Technology, Nanyang, China

**Keywords:** Gan-Mai-Da-Zao decoction, anxiolytic, elevated plus maze, light/dark box, GABA_A_ receptor, 5-HT_1A_ receptor

## Abstract

The Gan-Mai-Da-Zao (GMDZ) decoction is one of the most famous Chinese medicine prescriptions to treat emotional diseases in China. Here we examined the anxiolytic-like effects of the GMDZ decoction in mice. The mice were orally administered with GMDZ decoction (1, 2, and 4 g/kg, respectively) for 7 days, diazepam (2 mg/kg, p.o.) and buspirone (5 mg/kg, p.o.) were used as positive controls. Then, elevated plus maze (EPM) test, light/dark box (LDB) test, and marble burying (MB) test, open field (OF) test and rota-rod test were performed. We found that GMDZ treatment (2 and 4 g/kg) significantly increased the percentage of open arm entries and time spent on the open arms in EPM as compared to the control. GMDZ treatment also significantly increased the time spent in the light box and the number of light box entries in LDB and reduced the number of marbles buried in MB. Similarly to those observed with diazepam and buspirone. In contrast, GMDZ did not affect the locomotor activity in the OF and motor coordination in the rota-rod test. Furthermore, the anxiolytic-like effects induced by GMDZ were inhibited by the γ-aminobutyric acid-A (GABA_A_) receptor antagonist flumazenil and 5-hydroxytryptamine-1A (5-HT_1A_) receptor antagonist WAY-100635. These results showed that GMDZ possesses anxiolytic-like effects in animal models, and its mechanism of action might be modulated by 5-HT_1A_ and GABA_A_ receptors.

## Introduction

Anxiety disorders is one of the most common mental disorders that influence people of all ages in the general population and is becoming an increasing public health challenge worldwide (Mackenzie et al., 2011). The prevalence of anxiety disorders is more than 25% in the United States (Matsuzaki et al., 2012).Current therapies for anxiety disorders mainly consist of benzodiazepines and selective serotonin reuptake inhibitors (Girish et al., 2013). However, both these two types of drugs have well-known side effects (Pollack, 2002). Benzodiazepines have relation to muscle relaxation, sedation, and cognitive impairments; whereas, selective serotonin reuptake inhibitors show a delayed onset of action of several weeks (Shorter and Tyrer, 2003; Liu et al., 2015). Other problems can include the development of resistance to medicines and the risk of potential dependence (Mansouri et al., 2014). Therefore, the development of other antianxiety drugs with fewer side effects is necessary, which becomes one of the more pressing issues in the field of mental science (Harada et al., 2018).

Complementary and alternative therapies may play an important role in the clinical treatment of anxiety disorders (Hazim et al., 2014; Thorn et al., 2016). Compared to classical anxiolytic drugs, use of complementary and alternative medicine could show fewer side-effects (Li et al., 2016; Abouhosseini Tabari et al., 2018). The Gan-Mai-Da-Zao (GMDZ) decoction is one of the most famous herbal prescriptions in Chinese medical book Jin Gui Yao Lue (Medical Treasures of the Golden Chamber), which is written by medical sage Zhang Zhongjing (Ruan, 2003). The GMDZ decoction is comprised of three herbs *Triticum, Glycyrrhiza*, and *Zizyphi Fructus*. GMDZ decoction was used by Zhang Zongjing to treat Zang Zao syndrome, which is a kind of emotional diseases with symptoms such as a racing heart and shakiness. The contents about Zang Zao syndrome in Jin Gui Yao Lue were contained in anxiety of modern clinical diseases (Jun et al., 2014). Clinical studies showed that it is an effective anxiolytic prescription with good tolerance (Duan, 1996; Chen, 2014). However, the mechanisms underlying anxiolytic effect of GMDZ decoction remains unclear.

We previously found GMDZ decoction exerted anxiolytic effect in the open field (OF) test in chronic stressed rats (Lou et al., 2010). In this study, we further examined the anxiolytic-like effects of the GMDZ decoction in mice. The anxiolytic-like effects of the decoction were examined by the elevated plus maze (EPM), light/dark box (LDB), and marble burying (MB) tests, respectively. Moreover, to detect the locomotor activity and motor coordination, the OF and rota-rod tests were also conducted. Finally, GABA_A_ and 5-HT_1A_ receptors antagonists were used to clarify whether GABAergic and serotonergic systems were involved in GMDZ-induced anxiolytic-like effects.

## Materials and Methods

### Preparation of GMDZ Decoction

**FIGURE 1:**
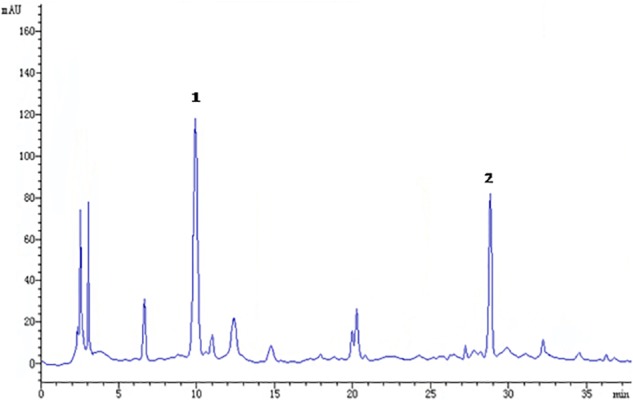
Chromatographic profile of Gan-Mai-Da-Zao (GMDZ) decoction; 1, liquiritin; 2, ammonium glycyrrhizinate. Chromatographic separation was achieved on a Kromasil 100-5 C18 column maintained at 25°C.

The raw herbs for GMDZ decoction were purchased from Tongrentang Pharmacy. The herb materials were authenticated by Prof. Zhao B, a professor of pharmacognostical identification in Beijing University of Chinese Medicine. The voucher specimens (D1312110715) were deposited in the storage cabinet of room 226 at the Institute of Psychology, Chinese Academy of Sciences. GMDZ extract was prepared according to our previous method with minor modifications (Lou et al., 2010). Briefly, three crude herbs (*Triticum aestivum* L., *Glycyrrhiza uralensis* Fisch., and *Ziziphus jujuba* Mill.) were mixed in a ratio of 3:2:3. Then the mixture was powdered and boiled in distilled water (40 g/320 mL, reflux, 2 h × 2). After completion of extraction, it was filtered and dried under reduced pressure at a temperature below 60°C. Quality control of GMDZ was performed by high-performance liquid chromatographic (HPLC) analysis (Figure 1). The contents of liquiritin and ammonium glycyrrhizinate in the extract were 11.3 mg/g and 5.2 mg/g. The dosages were presented in terms of the dried weight of the GMDZ decoction per unit body weight of the animals (g/kg).

### Animals and Treatment

Male ICR mice (18–22 g) were obtained from the Chinese Academy of Military Medical Sciences and kept in cages (25 × 15 × 14 cm) at 22 ± 1°C on a 12/12 h light/dark cycle (the light was on from 8:00 A.M. to 8:00 P.M.). The mice were housed five per cage, provided water and food *ad libitum*. All experiments were carried out in accordance with the National Institutes of Health Guide for Care and Use of Laboratory Animals and were approved by the Animal Care and Use Committee of the Institute of Psychology of the Chinese Academy of Sciences.

The present study was divided into two parts experiments. In the first experiment, mice were divided into six groups (*n* = 12): control, DZP, BUSP, GMDZ-1, GMDZ-2, and GMDZ-4. The diazepam (DZP, Yimin Pharmaceutical Factory, Beijing, China; 2 mg/kg) and buspirone (BUSP, PKU Healthcare corporation, Beijing, China, 5 mg/kg) were chosen as the positive control drugs, which were orally administrated for 7 days. GMDZ mice were orally administered GMDZ decoction (1, 2, and 4 g/kg, respectively) for 7 days, and control animals were orally administered the vehicle (saline) for 7 days. The behavior tests (EPM, LDB, MB, OF, and rota-rod test) were conducted 60 min after GMDZ administration or 30 min after DZP and BUSP administration at seventh day. Once the potential anxiolytic-like effect of GMDZ decoction was observed, another experiment was performed to investigate whether the GABA_A_ or 5-HT_1A_ receptor was involved in the anxiolytic effects of GMDZ decoction. Mice were divided into 10 groups (*n* = 12): Control, Flu, WAY, DZP, DZP+Flu, BUSP, BUSP+WAY, GMDZ, GMDZ+Flu, and GMDZ+WAY. The mice in DZP and DZP+Flu groups were received DZP (2 mg/kg, p.o.) for 7 days, the BUSP and BUSP+WAY groups were administrated with BUSP (5 mg/kg, p.o.) for 7 days, while GMDZ, GMDZ+Flu, and GMDZ+WAY received GMDZ (4 g/kg, p.o.) for 7 days. Control, Flu, and WAY animals were orally administrated the vehicle for 7 days. On the seventh day, flumazenil (Flu, 3 mg/kg; i.p.) was intraperitoneal injected 15 min before administration of DZP (DZP+Flu group), GMDZ (GMDZ+Flu group), and vehicle (Flu group), while WAY-100635 (WAY, 1 mg/kg, i.p.) was intraperitoneal injected 15 min before administration of BUSP (BUSP+WAY group), GMDZ (GMDZ+WAY group), and vehicle (WAY group). Then three behavior tests (EPM, LDB, and MB) were conducted 60 min after GMDZ administration or 30 min after DZP and BUSP administration. The dosages of GMDZ, DZP, WAY, and Flu were based on our previously studies (Lou et al., 2010; Liu et al., 2015), and the dose of BUSP 5 mg/kg is enough to produce an anxiolytic effect (Pires et al., 2013). As most Chinese medicines are slowly absorbed drugs, the behavior tests were conducted 60 min after GMDZ administration (Liu et al., 2015). DZP, BUSP, WAY, and Flu were all dissolved in physiological saline. The GMDZ decoctions were prepared with three concentrations (0.05, 0.1, and 0.2 g/ml) so that mice in GMDZ groups were all administrated orally in a volume of 0.5 ml/25 g body weight.

### Elevated Plus Maze

The maze was comprised of two open arms (30 × 5 × 0.2 cm) and two closed arms (30 × 5 × 15 cm) that directly opposed each other. The arms extended from a central platform (5 × 5 cm), and the entire apparatus was elevated 45 cm above the floor. A video camera was suspended above the maze to capture animals’ location in the maze. To begin with, the mouse was placed individually in the center of the maze facing an open arm, and the time spent on and the number of entries into and the open and closed arms were detected during a 5 min test period (Liu et al., 2015). An effective entry was defined as the placement of all four paws into an arm. The percent time spent in open arm [(open arm time/total time) × 100%] and open arm entries [(open arm entries/total arm entries) × 100%] were calculated for each animal. Heat maps were generated using the Noduls software (Netherland) to create a representative image of animal movement. The apparatus was cleanly wiped with 70% alcohol after each trial.

### Light/Dark Box

The light/dark apparatus is a rectangular box (45 × 21 × 21 cm) divided into two compartments, with one-third painted white and two-thirds painted black. The black compartment was closed with a lid, whereas white compartment was illuminated by two 60 W bulbs placed 30 cm above the box. These two compartments were separated by a divider with a 3.5 × 3.5 cm opening at floor level. The mouse was initially placed individually in the corner of the white compartment away from the dark compartment and observed for 5 min. The number of light box entries and time spent in the light compartment were detected (Narasingam et al., 2017). The apparatus was cleaned with 70% methanol between each test.

### Marble Burying Test

A normal glass cage (27 × 16 × 13 cm) with 25 glass marbles equidistantly distributed on a 5 cm layer of sawdust was used in this experiment. The animals were placed individually in the cages for 30 min. At the end of the test, the number of marbles buried in the sawdust was measured by an observer who was blinded to group assignment and outcome assessment. A marble was considered as hidden when it was at least two-thirds covered by bedding (Pires et al., 2013). After each test, the marbles and sawdust were washed and cleaned with ethanol 70%.

### Open Field Test

The OF device was comprised of a plexiglas arena (60 × 60 × 25 cm) with a white floor, which was divided into 36 squares (10 × 10 cm). For testing, a mouse was placed individually in the middle of the arena and the number of squares crossed (with four legs on each square) was recorded for 5 min (Rotheneichner et al., 2017). After each trial, the device was wiped clean with a 70% ethanol.

### Rota-Rod Test

Prior to experimentation, the animals were trained to learn the ability to remain for 180 s on a diameter rod with a rotation of 17 rpm. For testing, the animals were placed on the rotating bar, which is 2.5 cm in diameter and 25 cm above the floor. The number of falls and the time of spent on the rotating bar and were detected for a period of 180 s (de Almeida et al., 2012). After each trial, the apparatus was wiped clean with a70% ethanol.

### Statistical Analysis

The results were expressed as means ± SEM. Data were evaluated by one-way analysis of variance (ANOVA) with Dunnett’s tests for *post hoc* analysis. In the antagonistic experiments, two-way ANOVA followed by *post hoc* Bonferroni’s test was used. All data analyses were done by using GraphPad Prism 5.0. The threshold for statistical significance was set at *p* < 0.05.

## Results

### Effect of GMDZ Decoction in the Elevated Plus Maze

**FIGURE 2:**
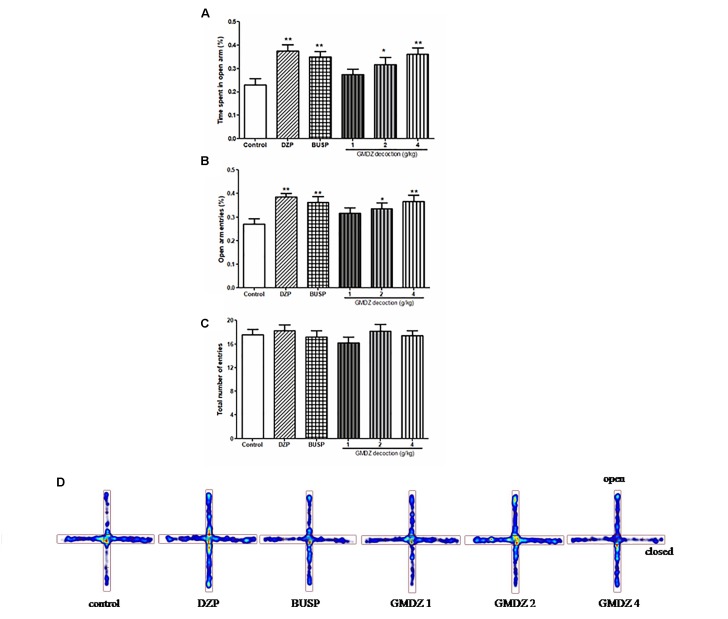
Effect of GMDZ decoction on the EPM test **(A)** The percentage of time spent on open arms. **(B)** The percent of open arms entries. **(C)** The total arm entries. **(D)** Representative heat map analysis of one animal in each group. Value are represented as mean ± SEM (*n* = 12). ^∗∗^*P* < 0.01 vs. control group. One way ANOVA with Student–Newman–Keuls *post hoc* test.

The one-way ANOVA indicated significant group effects in terms of the percent of time spent on the open arms [*F*_(5,66)_ = 4.301, *P* < 0.01; Figures 2A,D] and the percentage of open arm entries [*F*_(5,66)_ = 3.361, *P* < 0.01; Figure 2B]. GMDZ (2 and 4 g/kg) markedly increased the percentage of time spent on the open arms (*P* < 0.05 and *P* < 0.01, respectively) and the percent of open arm entries (*P* < 0.05 and *P* < 0.01, respectively) compared to control group treated with vehicle. DZP and BUSP treatment showed significant elevation in the percentage of time spent in the open arms (both *P* < 0.01) and open arm entries (both *P* < 0.01) as compared to control. No significant differences were observed in total number of entries among groups [*F*_(5,66)_ = 0.545, *P* > 0.05; Figure 2C]. The high dosage 4 g/kg was chosen for further antagonist experiments.

### Effects of Receptor Antagonists on the Anxiolytic-Like Effect of GMDZ Decoction in the Elevated Plus Maze

**FIGURE 3:**
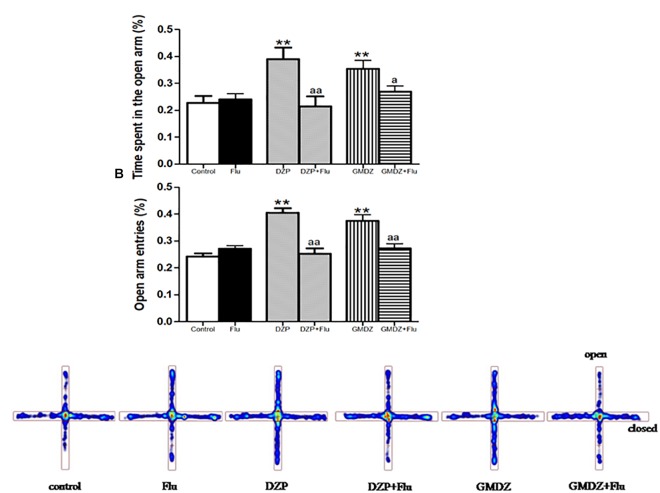
Effects of receptor antagonists on anxiolytic-like effect of GMDZ in EPM test. **(A)** Effect of Flu on the percentage of time spent in open arms. **(B)** Effect of Flu on the percent of open arms entries. **(C)** Representative heat map analysis of one animal in each group. Value are represented as mean ± SEM (*n* = 12). ^∗∗^*P* < 0.01 vs. control group; ^a^*P* < 0.05 or ^aa^*P* < 0.01 treatment vs. antagonist (interaction effect). Two-way ANOVA with Bonferroni *post hoc* test.

**FIGURE 4:**
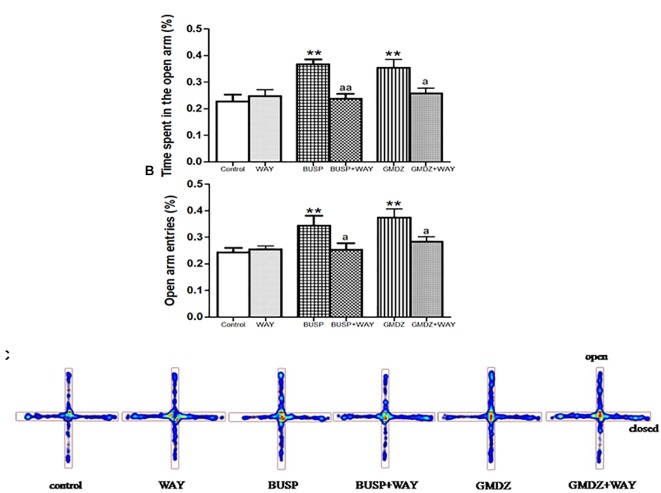
Effects of receptor antagonists on anxiolytic-like effect of GMDZ in EPM test. **(A)** Effect of WAY on the percentage of time spent in open arms. **(B)** Effect of WAY on the percent of open arms entries. **(C)** Representative heat map analysis of one animal in each group. Value are represented as mean ± SEM (*n* = 12). ^∗∗^*P* < 0.01 vs. control group; ^a^*P* < 0.05 or ^aa^*P* < 0.01 treatment vs. antagonist (interaction effect). Two-way ANOVA with Bonferroni *post hoc* test.

Flu was co-administrated with DZP or GMDZ to determine whether the GABA_A_-benzodiazepine receptor antagonist would alter the anxiolytic-like effect in EPM. DZP and GMDZ treatment showed a significant increase in the percentage of time spent on open arms (both *P* < 0.01) and the percent of open arms entries (both *P* < 0.01) compared to the vehicle group, while Flu alone was not markedly different from the vehicle group (both *P* > 0.05). As shown in Figures 3A–C, the two way ANOVA revealed that Flu could antagonize the DZP effect on the percentage of time spent on open arms [DZP (treatment): *F*_(1,44)_ = 4.38, *P* < 0.05; Flu (antagonist): *F*_(1,44)_ = 4.16, *P* < 0.05; and DZP (treatment) × Flu (antagonist) interaction: *F*_(1,44)_ = 8.16, *P* < 0.01] and the percent of open arms entries [DZP: *F*_(1,44)_ = 10.01, *P* < 0.01; Flu: *F*_(1,44)_ = 5.38, *P* < 0.05; and DZP × Flu interaction: *F*_(1,44)_ = 15.7, *P* < 0.01], and it also could inhibit the GMDZ effect in the percentage of time spent on open arms [GMDZ: *F*_(1,44)_ = 4.62, *P* < 0.05; Flu: *F*_(1,44)_ = 1.93, *P* > 0.05; and GMDZ × Flu interaction: *F*_(1,44)_ = 4.15, *P* < 0.05], and the percent of open arms entries [GMDZ: *F*_(1,44)_ = 7.63, *P* < 0.01; Flu: *F*_(1,44)_ = 2.27, *P* > 0.05; and GMDZ × Flu interaction: *F*_(1,44)_ = 7.18, *P* < 0.01]. WAY was then co-administration with BUSP or GMDZ to investigate if 5-HT_1A_ receptor antagonist could affect the anxiolytic-like effect. The two-way ANOVA showed BUSP and GMDZ significantly increased the percentage of time spent in open arms (both *P* < 0.01) and the percent of open arm entries (both *P* < 0.01) compared with vehicle group (Figures 4A–C), the WAY alone was not significantly different from vehicle (both *P* > 0.05). WAY could antagonize the BUSP effect on the percent of time spent on open arms [BUSP: *F*_(1,44)_ = 8.38, *P* < 0.01, WAY: *F*_(1,44)_ = 3.21, *P* > 0.05, and BUSP × WAY interaction: *F*_(1,44)_ = 11.15, *P* < 0.01] and the percent of open arms entries [BUSP: *F*_(1,44)_ = 4.06, *P* < 0.05, WAY: *F*_(1,44)_ = 2.56, *P* > 0.05, and BUSP × WAY interaction: *F*_(1,44)_ = 4.4, *P* < 0.05], and it also could inhibit the GMDZ effect in the percentage of time spent on open arms [GMDZ: *F*_(1,44)_ = 6.84, *P* < 0.05, WAY: *F*_(1,44)_ = 2.11, *P* > 0.05, and GMDZ × WAY interaction: *F*_(1,44)_ = 5.13, *P* < 0.05], and the number of open arms entries [GMDZ: *F*_(1,44)_ = 13.18, *P* < 0.01, WAY: *F*_(1,44)_ = 3.16, *P* > 0.05, and GMDZ × WAY interaction: *F*_(1,44)_ = 5.55, *P* < 0.05].

### Effect of GMDZ Decoction in the Light/Dark Box Test

**FIGURE 5:**
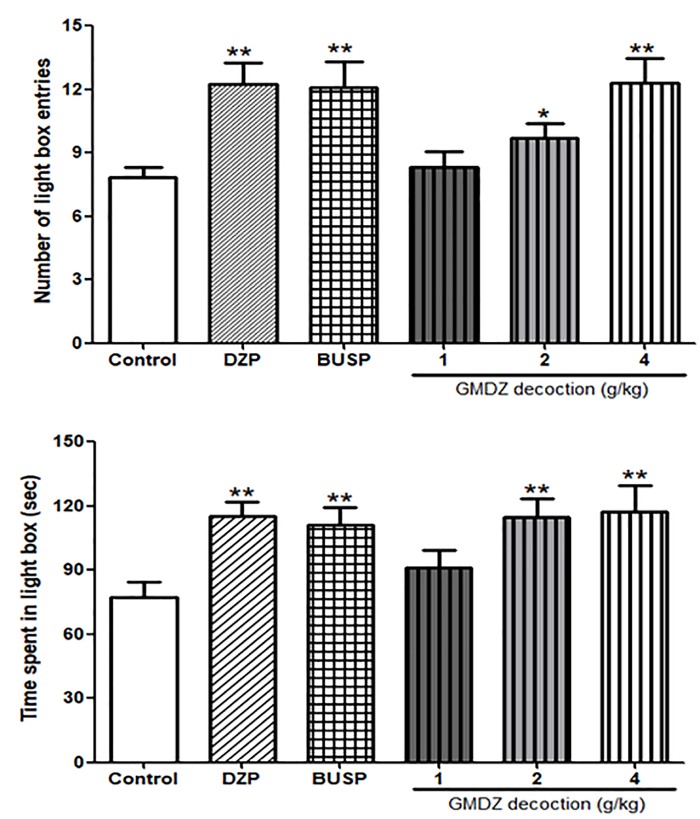
Effect of GMDZ decoction on the LDB test. **(A)** The numbers of light box entries. **(B)** The time spent in the light box. Value are represented as mean ± SEM (*n* = 12). ^∗^*P* < 0.05 or ^∗∗^*P* < 0.01 vs. control group. One way ANOVA with Student–Newman–Keuls *post hoc* test.

As shown in Figures 5A,B, the one-way ANOVA showed significant differences among six groups in the number of light box entries [*F*_(5,66)_ = 4.905, *P* < 0.01] and time spent in the light box [*F*_(5,66)_ = 3.471, *P* < 0.01]. Compared with the control group, treatment with GMDZ at dosages of 2 and 4 g/kg significantly increased the number of light box entries (*P* < 0.05 and *P* < 0.01, respectively) and time spent in the light box (both *P* < 0.01). Both DZP and BUSP treatment also significantly elevated the number of light box entries (both *P* < 0.01) and the time spent in the light box (both *P* < 0.01).

### Effects of Receptor Antagonists on the Anxiolytic-Like Effect of GMDZ in the Light/Dark Box Test

**FIGURE 6:**
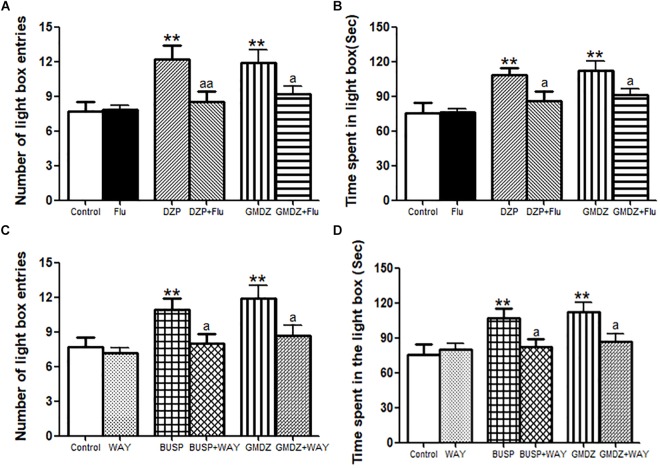
Effect of receptor antagonists on the anxiolytic-like effect of GMDZ in the LDB test. **(A)** Effect of Flu on the numbers of light box entries. **(B)** Effect of Flu on the time spent in the light box. **(C)** Effect of WAY on the numbers of light box entries. **(D)** Effect of WAY on the time spent in the light box. Value are represented as mean ± SEM (*n* = 12). ^∗∗^*P* < 0.01 vs. control group; ^a^*P* < 0.05 or ^aa^*P* < 0.01 treatment vs. antagonist (interaction effect). Two-way ANOVA with Bonferroni *post hoc* test.

As shown in Figures 6A,B, Flu was i.p. administrated to determine whether it could block the anxiolytic-like effect of DZP or GMDZ in LDB. The statistical analyses revealed that DZP and GMDZ significantly increased the number of light box entries (both *P* < 0.01) and time spent in the light box (both *P* < 0.01) compared with the vehicle group, while Flu alone was not markedly different from the vehicle group (both *P* > 0.05). Flu could antagonize the DZP effect on the number of light box entries [DZP: *F*_(1,44)_ = 8.21, *P* < 0.01; Flu: *F*_(1,44)_ = 2.37, *P* > 0.05; and DZP × Flu interaction: *F*_(1,44)_ = 7.50, *P* < 0.01] and time spent in the light box [DZP: *F*_(1,44)_ = 10.26, *P* < 0.01; Flu: *F*_(1,44)_ = 2.61, *P* > 0.05; and DZP × Flu interaction: *F*_(1,44)_ = 3.96, *P* < 0.05], and it also could block the GMDZ effect on the number of light box entries [GMDZ: *F*_(1,44)_ = 12.07, *P* < 0.01; Flu: *F*_(1,44)_ = 2.58, *P* > 0.05; and GMDZ × Flu interaction: *F*_(1,44)_ = 4.12, *P* < 0.05], and time spent in the light box [GMDZ: *F*_(1,44)_ = 14.53, *P* < 0.01; Flu: *F*_(1,44)_ = 2.38, *P* > 0.05; and GMDZ × Flu interaction: *F*_(1,44)_ = 3.95, *P* < 0.05]. WAY was pretreated with BUSP or GMDZ to investigate if 5-HT_1A_ receptor was involved in the anxiolytic-like of GMDZ. The two-way ANOVA showed BUSP and GMDZ significantly increased the number of light box entries (both *P* < 0.01) and time spent in the light box (both *P* < 0.01) as compared to vehicle group (Figures 6C,D), the WAY alone was not significantly different from vehicle (both *P* > 0.05). WAY could antagonize the BUSP effect on the number of light box entries [BUSP: *F*_(1,44)_ = 6.37, *P* < 0.05; WAY: *F*_(1,44)_ = 3.04, *P* > 0.05; and BUSP × WAY interaction: *F*_(1,44)_ = 4.98, *P* < 0.05] and time spent in the light box [BUSP: *F*_(1,44)_ = 5.19, *P* < 0.05; WAY: *F*_(1,44)_ = 1.88, *P* > 0.05; and BUSP × WAY interaction: *F*_(1,44)_ = 4.08, *P* < 0.05], and it also could inhibit the GMDZ effect on the number of light box entries [GMDZ: *F*_(1,44)_ = 11.14, *P* < 0.01; WAY: *F*_(1,44)_ = 3.14, *P* > 0.05; and GMDZ × WAY interaction: *F*_(1,44)_ = 3.99, *P* < 0.05], and time spent in the light box [GMDZ: *F*_(1,44)_ = 9.05, *P* < 0.01; WAY: *F*_(1,44)_ = 2.22, *P* > 0.05; and GMDZ × WAY interaction: *F*_(1,44)_ = 5.43, *P* < 0.05].

### Effect of GMDZ Decoction in the Marble Burying Test

**FIGURE 7:**
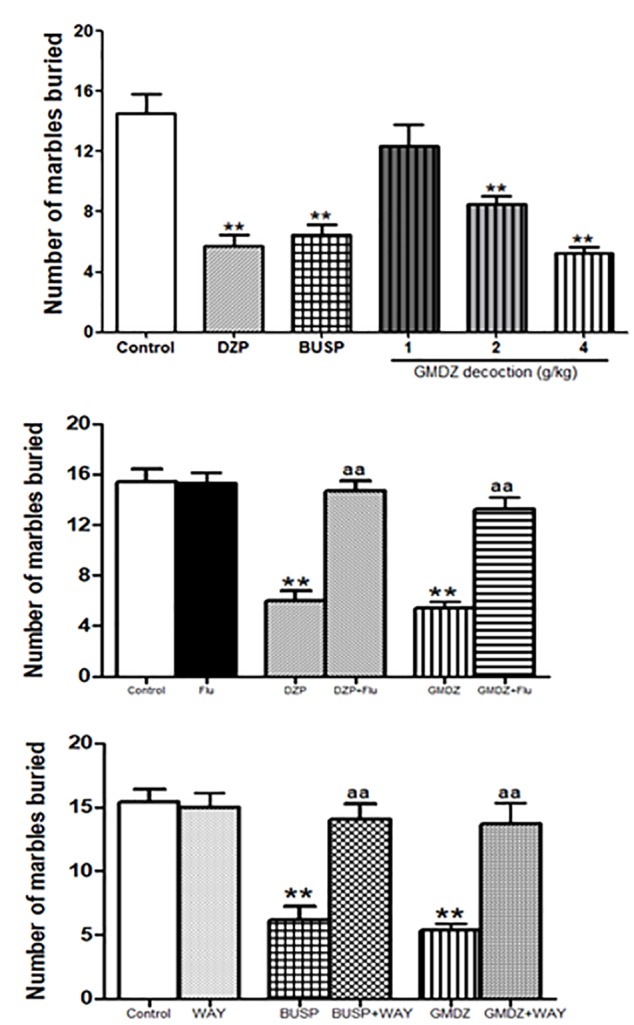
Effect of GMDZ decoction and receptor antagonist on the number of marbles buried in the MB test. **(A)** Effect of GMDZ decoction on the MB test. **(B)** Effect of Flu on the numbers of marbles buried. **(C)** Effect of WAY on the number of marbles buried. Value are represented as mean ± SEM (*n* = 12). ^∗∗^*P* < 0.01 vs. control group; One way ANOVA with Student–Newman–Keuls *post hoc* test. ^a^*P* < 0.05 or ^aa^*P* < 0.01 treatment vs. antagonist (interaction effect). Two-way ANOVA with Bonferroni *post hoc* test.

There were significant group effects in the number of marbles buried among groups as analyzed by a one-way ANOVA analysis [*F*_(5,66)_ = 16.545, *P* < 0.01, Figure 7A]. GMDZ at dosages of 2 and 4 g/kg significantly reduced the number of marbles buried (both *P* < 0.01), similar effect was also observed in DZP and BUSP groups (both *P* < 0.01).

### Effect of Antagonist on the Anxiolytic-Like Effect of GMDZ Decoction in the Marble Burying Test

The mice were pretreated with Flu before administration of DZP or GMDZ. As shown in Figure 7B, DZP and GMDZ significantly increased the number of marbles buried (both *P* < 0.01) compared to the vehicle group, while the Flu alone was not significantly different from the vehicle group (*P* > 0.05). The two way ANOVA showed that pretreatment with Flu before the DZP administration could antagonize the drug effects [DZP: *F*_(1,44)_ = 33.01, *P* < 0.01; Flu: *F*_(1,44)_ = 13.85, *P* < 0.05; and DZP × Flu interaction: *F*_(1,44)_ = 25.76, *P* < 0.01], and it also could inhibit the GMDZ effect [GMDZ: *F*_(1,44)_ = 50.51, *P* < 0.01; Flu: *F*_(1,44)_ = 10.62, *P* < 0.01; and GMDZ × Flu interaction: *F*_(1,44)_ = 22.45, *P* < 0.01]. We further explored whether WAY could affect the effect of BUSP and GMDZ. The two-way ANOVA showed BUSP and GMDZ significantly increased the number of marbles buried as compared to control group (both *P* < 0.01, Figure 7C), the WAY alone was not significantly different from control (*P* > 0.05).The number of marbles buried was significantly decreased when WAY was injected before BUSP treatment [BUSP: *F*_(1,44)_ = 21.47, *P* < 0.01; WAY: *F*_(1,44)_ = 8.68, *P* < 0.01; and BUSP × WAY interaction: *F*_(1,44)_ = 14.42, *P* < 0.01], and it was also reduced by WAY treatment before GMDZ administration [GMDZ: *F*_(1,44)_ = 24.92, *P* < 0.01; WAY: *F*_(1,44)_ = 6.56, *P* < 0.05; and GMDZ × WAY interaction: *F*_(1,44)_ = 14.57, *P* < 0.01].

### Effects of GMDZ Decoction in the Open Field Test

As shown in Table 1, one-way ANOVA showed significant differences in the number of squares crossed [*F*_(5,66)_ = 4.25, *P* < 0.01]. Administration with DZP significantly reduced locomotor activity as compared to control group (*P* < 0.01). BUSP treatment slightly decrease the locomotor activity, but the difference was not statistically significant (*P* > 0.05). All GMDZ groups did not affect the locomotor activity as compared to control (*P* > 0.05).

**Table 1:** Effects of GMDZ decoction on the open field test in mice.

Groups	Dose	Number	Number of squares crossed
Control	saline	12	71.26 ± 5.39
DZP	2 mg/kg	12	46.26 ± 3.18^∗∗^
BUSP	5 mg/kg	12	59.88 ± 3.84
GMDZ	1 g/kg	12	71.26 ± 4.92
GMDZ	2 g/kg	12	67.92 ± 5.95
GMDZ	4 g/kg	12	70.15 ± 4.86

*Values are expressed as mean ± SEM (n = 12). ^∗∗^P < 0.01 vs. control group. One way ANOVA with Student–Newman–Keuls post hoc test.*

### Effect of GMDZ Decoction on the Rota-Rod Test

**FIGURE 8:**
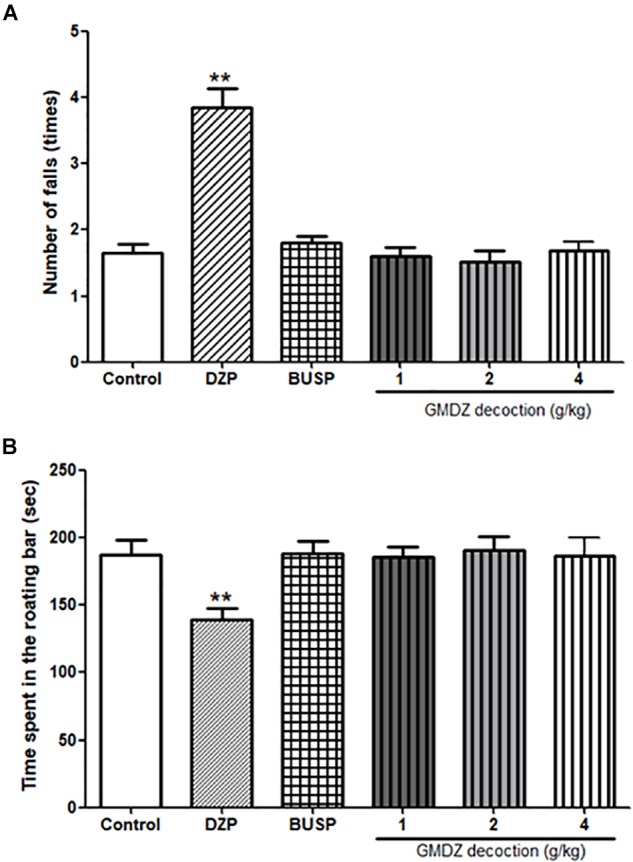
Effect of GMDZ decoction on the rota-rod test. **(A)** Number of falls. **(B)** Time spent on the rotating bar. Value are represented mean ± SEM (*n* = 12). ^∗∗^*P* < 0.01 vs. control group. One way ANOVA with Student–Newman–Keuls *post hoc* test.

A one-way ANOVA revealed significant differences on the number of falls [*F*_(5,66)_ = 28.113, *P* < 0.01, Figure 8A] and the time spent on the rotating bar [*F*_(5,66)_ = 3.763, *P* < 0.01, Figure 8B]. Compared with the control group, DZP treatment significantly increased the number of falls by 2.3 fold (*P* < 0.01), while the GMDZ groups and BUSP did not affect the number of falls (all *P* > 0.05). The time spent in the rotating bar was reduced in DZP group (77%) in compared to control but did not change in the GMDZ groups and BUSP (*P* > 0.05).

## Discussion

Gan-Mai-Da-Zao decoction is a famous Chinese herb medicine that is widely used in East Asia for emotional diseases, e.g., depression and anxiety. Known as Ganmckdaecko-tang in Korea and Kambakutaisoto in Japan, GMDZ is an effective prescription in treating depression (Jun et al., 2014; Kim et al., 2017). GMDZ decoction may possess anxiolytic activity because it increased the number of rearings in OF test in stressed rats (Lou et al., 2010). Here we further investigated the anxiolytic-like effects of GMDZ decoction using a battery of behavioral tests (EPM, LDB, and MB). DZP and BUSP were used as reference anxiolytics. Consistent with previous research (Lou et al., 2010), this study showed that GMDZ decoction exerted anxiolytic-like actions in mice and no major side effects, and the mechanism might be related with its action on benzodiazepine and 5-HT receptors.

GABAergic and serotonergic neurotransmission are considered to play important roles in the regulation of anxiety (Narasingam et al., 2017). Previous researches showed that reduced brain levels of the inhibitory neurotransmitter GABA and its major receptor, GABA_A_ receptor, are involved in the etiology of anxiety (Luscher et al., 2011). The classical benzodiazepines can relieve anxiety, while GABA_A_ receptor antagonists induce anxiety (Dalvi and Rodgers, 1996; Chioca et al., 2013). Besides, altered 5-HT signal pathway contributes to the anxiety (Mi et al., 2017). The 5-HT_1A_ receptor is widely distributed in the frontal cortex, amygdale and hippocampus, and activation of 5-HT_1A_ receptor could decrease 5-HT outflow and reduce serotonergic neuron activity (Zhang et al., 2016). Thus, 5-HT_1A_ receptor is of great important in modulating anxiety-related behavior and might offer the potential to regulate anxiety (Jung et al., 2013). In order to investigate the mechanism underlying anxiolytic-like effect of GMDZ, a pharmacological study using GABA_A_ receptor antagonist and 5-HT_1A_ receptor antagonist was conducted in this study.

The EPM has been used effectively to evaluate the efficacy of anxiety modifying interventions and investigate the neurobiological basis of anxiety disorders (Walf and Frye, 2007). Exposure to the open arms of the maze causes markedly more anxiety-related behaviors than exposure to the closed arms (Ludwig et al., 2008). An elevation in the number of open arm entries and the time spent in open arm is a very powerful indicator for the anxiolytic agent (Wilson et al., 2013; Mansouri et al., 2014). In this study, we found that GMDZ treatment significantly increased the percent of time spent in open arms and the percent of arm entries in a dose-dependent manner, indicating that GMDZ decoction can produce anxiolytic-like effects in mice. These effects could be antagonized by the selective 5HT_1A_ receptor antagonist, WAY and GABA_A_ receptor antagonist, Flu.

To further demonstrate the potential anxiolytic-like effects of GMDZ decoction, the LDB test was also conducted. This experiment is based on the inherent aversion of animals to brightly illuminated areas and on the spontaneous exploratory behavior of animals respond to mild stressors, such as light and novel circumstance (Li et al., 2003; Fortes et al., 2013).Thus, the time spent in the light part of the box is the most useful marker for investigating the anxiolytic action (Moniruzzaman et al., 2018). Our data clearly showed that GMDZ decoction significantly increased the time spent in light box and the number of light box entries in a dose-dependent manner. Similarly, the anxiolytic-like effect in LDB test was also blocked by Flu and WAY.

Although the good predictive validity of the EPM and LDB tests for anxiolytic drugs, the MB test was also added to avoid false-positive results. As anxiolytic agents could decrease the number of marbles buried in this test, without inducing any changes in locomotor activity (Gaikwad and Parle, 2011). GMDZ decoction markedly decreased the number of marbles buried in a dose-dependent manner, and the anxiolytic-like effect was also inhibited by Flu and WAY.

The EPM is based on exploration behavior, but this test is also dependent on the motor activity of the rodents. Therefore, the locomotor activity should be detected to interpret drug effects as being specific to anxiety-related behavior (Chassot et al., 2011). The total arm entries were measured as an marker of the locomotor activity in EPM (Walf and Frye, 2007). GMDZ decoction did not alter the total arm entries, suggesting that the anxiolytic-like effects in EPM were not due to a stimulation of locomotor activity. This was further confirmed by the OF test, which is widely used to evaluate the locomotor activity and anxiety. GMDZ treatment did not exhibit alterations in the number of squares crossed in the OF. These results clearly showed that GMDZ treatment does not affect the locomotor activity in mice.

Diazepam is a GABA_A_ receptor agonist and BUSP is a partial 5-HT_1A_ receptors agonist (Belmer et al., 2018). In line with past studies (Pires et al., 2013; Liu et al., 2015), the current findings confirmed the anxiolytic effect of DZP and BUSP. As DZP and BUSP increased the percentage of time spent and the number of entries into open arms, elevated the number of light box entries and the time spent in light box, and reduce the number of marbles buried in EPM, LDB, and MB tests, respectively. Pretreated with Flu reversed the anxiolytic-like effects of DZP, whereas WAY blocked the anxiolytic-like effects of BUSP. Moreover, we found that BUSP slight decreased the locomotor activity in OF test, which might be because activation of 5HT_1A_ receptor can lead to neural inhibition (Muller et al., 2007). Besides its anxiolytic effect, the 5HT_1A_ receptor agonist has profound effect on memory or reward (Shapiro et al., 2018).

The benzodiazepines (e.g., DZP) and barbiturates can cause muscle weakness and sedation (Akindele et al., 2013). Rota-rod test was therefore conducted to evaluate the motor coordination and muscle relaxation, which could be observed by the time of permanence and number of falls on the rotating rods. The present study suggested that DZP reduced the time of permanence on rotating rod and increased the number of falls in rota-rod test, indicating a sedation effect that influenced the motor coordination. Contrary to DZP, GMDZ decoction, at the doses employed, did not exhibit significant effect on motor coordination in rota-rod test.

Gan-Mai-Da-Zao decoction had stable anxiolytic-like effects, and WAY or Flu could inhibit the anxiolytic-like effect of GMDZ in EPM, LDB, and MB. However, we did not measure the combination effects of these two antagonists. As Flu blocks GABAergic system and WAY inhibits the serotonergic system, Flu and WAY together might have greater influence on the anxiolytic effects, and it will be detected in a future study. In addition, the levels of GABA and monoamine in brain were not measured in this study, and the effects of GMDZ on changes of GABA and serotonin pathways remain to be further investigated.

## Conclusion

In summary, the present data show that GMDZ decoction possesses strong anxiolytic-like effects in the EPM, LDB, and OF tests but did not alters locomotor activity and motor coordination. The antagonism experiments suggest that the mode of action for GMDZ is via the GABAergic and serotonergic systems.

## Author Contributions

J-YG designed the study and drafted the manuscript. L-JG, H-SC, and Y-XY conceived of the study. All authors read and approved the final manuscript.

## Conflict of Interest Statement

The authors declare that the research was conducted in the absence of any commercial or financial relationships that could be construed as a potential conflict of interest.
